# Maintenance of the Cell Morphology by MinC in *Helicobacter pylori*

**DOI:** 10.1371/journal.pone.0071208

**Published:** 2013-08-01

**Authors:** Pei-Yu Chiou, Cheng-Hung Luo, Kai-Chih Chang, Nien-Tsung Lin

**Affiliations:** 1 Institute of Medical Sciences, Tzu Chi University, Hualien, Taiwan; 2 Department of Laboratory Medicine and Biotechnology, Tzu Chi University, Hualien, Taiwan; 3 Department of Microbiology, Tzu Chi University, Hualien, Taiwan; Indian Institute of Science, India

## Abstract

In the model organism *Escherichia coli*, Min proteins are involved in regulating the division of septa formation. The computational genome analysis of *Helicobacter pylori*, a gram-negative microaerophilic bacterium causing gastritis and peptic ulceration, also identified MinC, MinD, and MinE. However, MinC (HP1053) shares a low identity with those of other bacteria and its function in *H. pylori* remains unclear. In this study, we used morphological and genetic approaches to examine the molecular role of MinC. The results were shown that an *H. pylori* mutant lacking MinC forms filamentous cells, while the wild-type strain retains the shape of short rods. In addition, a *minC* mutant regains the short rods when complemented with an intact *minC_Hp_* gene. The overexpression of MinC_Hp_ in *E. coli* did not affect the growth and cell morphology. Immunofluorescence microscopy revealed that MinC_Hp_ forms helix-form structures in *H. pylori*, whereas MinC_Hp_ localizes at cell poles and pole of new daughter cell in *E. coli*. In addition, co-immunoprecipitation showed MinC can interact with MinD but not with FtsZ during mid-exponential stage of *H. pylori*. Altogether, our results show that MinC_Hp_ plays a key role in maintaining proper cell morphology and its function differs from those of MinC_Ec_.

## Introduction

*Helicobacter pylori*, the etiologic agent of human gastritis, peptic ulceration, and gastric carcinoma, infects at least half of the world’s population with the organism being highly restricted to the gastric mucosa of humans [1]. During infection, the major actively replicating forms of *H. pylori* cells are spiral-shaped, but they can convert to cocci under environmental stresses, such as starvation and antibiotic treatment. The coccoid form is viable, but not culturable in vitro. It is less virulent than the spiral form; however, it is thought to be crucial in disease transmission and insensitive to antibiotic treatment [2]. Therefore, cell shape is an important pathogenicity factor for *H. pylori*. So far, the maintenance and establishment of the spiral structure in *H. pylori* is known to occur through peptidoglycan relaxation and an intracellular scaffold [3], [4], [5], [6]. While cell division accuracy is crucial for maintaining the shape of some bacteria [7], little is known for *H. pylori*.

The process of cell division involves the spatial and temporal regulation of the septum formation by the cytoskeletal proteins [7]. In *Escherichia coli*, precise formation of septum between the two newly segregated sister chromosomes is initiated by FtsZ, which assembles into a ring and recruits a number of proteins, such as FtsA, ZipA, and ZapA, to form the septal ring [8]. In addition, Min proteins are required for the correct division site selection, which prevent polar divisions by blocking the Z ring assembly at cell poles [8]. The functions of FtsZ and *min* operon have been characterized in many bacteria [9], [10], [11], [12], [13]. Although the task of these proteins, which are involved in cell division, is almost identical in reported bacteria, the components and precise regulation mechanisms in preventing polar division of Min systems appears to be different among prokaryotic cells. For example, the Min system, which consists of 3 proteins, MinC, MinD, and MinE, and which is composed of an operon in *E. coli*, can oscillate periodically between the 2 poles of a cell, and MinE destabilized, but only 2 of them, MinC and MinD, are present in *Bacillus subtilis*. The function of MinE is substituted by an unrelated DivIVA protein, which targets MinCD in division sites and retains them at the cell poles. However, we found that the homologs of *minC*, *D*, and *E* are present, but not contained, in an operon in the sequenced genomes of *H. pylori*. Moreover, the amino acid sequences of MinC show a low identity with the corresponding proteins of reported bacteria. So far, whether *minC* plays any role in cell division of *H. pylori* remains unclear. Therefore, studying MinC’s functions is vital for understanding the cell division and shape-determining factors of *H. pylori*. In this study, we generated a *minC* mutant to study its biological characters. Our results show that *minC* plays a crucial role in maintaining the cell morphology and the movement capabilities of *H. pylori*.

## Materials and Methods

### Bacterial Strains, Plasmids and Growth Conditions

Bacterial strains and plasmids used in this study are listed in Table 1. Strains of *E. coli* were cultivated in Luria-Bertani (LB) (Difco Laboratories, Detroit, MI) solid and liquid media at 37°C. *H. pylori* NCTC 11637 was used to construct mutants. *H. pylori* strains were grown microaerobically at 37°C on blood agar plate (BAP) containing Columbia agar base (Becton Dikinson, Franklin Lakes, NJ, USA) and 5% horse blood or in Brucella broth (Becton Dikinson) containing 5% fetal bovine serum (FBS). Bacterial growth was measured by monitoring OD_600_, while live cells were determined by viable count on BAPs. When required, antibiotics were supplemented: ampicillin (Ap, 100 µg/mL), chloramphenicol (Cm, 30 µg/mL), and kanamycin (Kan, 50 µg/mL).

**Table 1:** Strains and plasmids used in this study.

Strain or plasmid	Genotype or description	Source
***Strains***		
*E.coli*		
Top10	F^-^mcrΔ (mrr-hsdRMS-mcrBC) 80*lac*ZΔM15 *lac*XΔ74 *rec*A1 *deo*R *ara*D139Δ (*ara-leu*) 7697 *gal*U *gal*K*rps*L (Str^R^) *end*A1 *nup*G	Invitrogen
BL21(DE3)	F^–^ *dcm ompT hsdS* (r_B_^–^ m_B_^–^) *gal* λ(DE3)	Stratagene
MG1655	wild-type	[30]
*H. pylori*		
NCTC 11637	wild-type, containing the entire *minC*	ATCC 43504
PY1	11637, *minC*::*cat*	This study
PY2	PY1, *hp0405*::P*_flaA_-minC_Hp_ kan*	This study
PY2-5	PY1, *hp0405*::P*_flaA_-minC_Ec_ kan*	This study
PY3	11637, *hp0405*::P*_flaA_-minC_Hp_ kan*	This study
PY3-1	11637, *hp0405*::P*_flaA_-minC_Ec_ kan*	This study
***Plasmids***		
pAV35	chloramphenicol acetyltransferase (*cat*) cassette; Cm^r^	[18]
pJMK30	kanamycin resistance (*kan*) cassette; Kan^r^	[18]
pUC18	Cloning vector, Ap^r^	Fermentas
pOC10	Cloning vector derived from pOK12, replacement of the *kan* gene with the *cat* gene from pAV35, Cm^r^	[19]
pTZ57R/t	T/A Cloning vector, Ap^r^	Fermentas
pET30a	Cloning and expression vector, Kan^r^	Novagen
pBAD33	pBAD expression; pACYC184 ori; Cm^r^	[31]
pCHL2	*hp0405*::P*_flaA_ kan*_,_ Kan^r^, Cm^r^	This study
pCPY001	pUC18 containing *minC_Hp_*, Ap^r^	This study
pCPY002	pCPY001 with *cat* inserted into the unique SphI site of *minC_Hp_*, Ap^r^, Cm^r^	This study
pCPY003	pCHL2 containing *minC_Hp_* under *flaA* promoter, *hp0405*::P*_flaA_-minC_Hp_ kan*_,_ Kan^r^, Cm^r^	This study
pCPY004	pET30a containing 6×*his*-*minC_Hp_*_,_ Kan^r^	This study
pCPY005	pET30a containing 6×*his*-*minC_Ec_*_,_ Kan^r^	This study
pCPY006	pCHL2 containing *minC_Ec_* under *flaA* promoter, *hp0405*::P*_flaA_-minC_Ec_ kan*_,_ Kan^r^, Cm^r^	This study
pCPY007	pET30a containing 6×*his*-*ftsZ*_,_ Kan^r^	This study
pCPY008	pET30a containing 6×*his*-*minD*_,_ Kan^r^	This study
pCPY009	pBAD33 containing *minC_Hp_*, Cm^r^	This study
pCPY010	pBAD33 containing *minC_Ec_*, Cm^r^	This study
pOC0405	pOC10 containing *hp0405*, Cm^r^	This study
pTZ-PflaA	pTZ57R/t containing *H. pylori* flagella promoter, Ap^r^	This study
pTZ-PflaAKm	pTZ-PflaA containing *kan* from pJMK30, Ap^r^, Kan^r^	This study

### DNA Techniques

The methods described by Sambrook et al [14] were used for preparation of chromosomal DNAs, restriction digestion, DNA ligation and *E. coli* transformations. Plasmids were isolated by using High-Speed Plasmid Mini Kit (Geneaid, Taipei, Taiwan). Natural transformation of *H. pylori* was performed as described elsewhere [15], [16].

### Cell Length Determination, Immunostaining, and Image Acquisition

*H. pylori* from overnight liquid cultures was inoculated into fresh Brucella broth to obtain an initial OD_600_ of 0.05 and grown to an OD_600_ of 0.6 to 0.8. Cells were examined microscopically on poly L-lysine-treated slides with a thin layer of 1% agarose in LB. Cell length was measured as the axis length from one pole to the other of the cells captured in microscope, using ImageJ version 1.46 (http://rbs.info.nih.gov/ij/). Average cell length was determined using at least 2 independent measurements, each on 200 cells. DNA was stained with 4′, 6-diamidino-2-phenylindole (DAPI; Sigma, St. Louis, MO) at a final concentration of 1 µg/mL and membrane was stained with FM4-64 (Molecular Probes/Invitrogen) at a concentration of 1 µg/mL. Bacterial viability was determined by staining the cells with SYTO9/propidium iodine (PI) (LIVE/DEAD BacLight kit, Molecular Probes/Invitrogen) at 24, 48, and 72 h, followed by fluorescence microscopic observation of the stained cells. Images were obtained with a Nikon E800 microscope using a 100×Objective with A = 1.45 and processed using Adobe Photoshop CS3.

The subcellular localization of MinC_Hp_ was carried out using immunofluorescence (IF) microscopy [17]. Bacteria were spread on a clean glass slide and allowed to dry briefly. Bacteria on the glass slides were fixed with methanol at room temperature for 15 min, followed by incubation with 0.1% Triton X-100 in PBS for 1 h. The bacteria were treated with 100 µg/mL of lysozyme and 5 mM EDTA in PBS for 1 h at room temperature. Prior to IF staining, bacteria were incubated with 10% (w/v) bovine serum albumin (BSA) in PBS for 30 min at 37°C to block nonspecific binding. Three PBS washes were performed following each incubation or treatment. After incubation for 1 h with anti-MinC_Hp_ (1∶200), the slides were washed 5 times with PBS containing 0.05% Tween 20 (PBST). Incubation using FITC-conjugated anti-rabbit IgG (1∶500) (Santa Cruz, CA, USA) diluted in blocking buffer was carried out for 30 min at 37°C. The cells were washed 3 times with PBST. The nucleoids were stained with DAPI at a final concentration of 0.5 µg/mL in H_2_O. The cells were washed once in H_2_O. The images of the bacteria were subsequently visualized with a Nikon E800 microscopy.

### Sequencing and Identification of the *minC* Gene

The oligonucleotide primers used in this study are listed in Table 2. Primers HP1054-F and HP1052-R for a PCR corresponded to the nucleotide (nt) −924 to −946, relative to the *hp1054* start codon, and nt −269 to −248, relative to the termination codon of *hp1052*, respectively. A PCR was performed to amplify the fragment, using the *H. pylori* NCTC 11637 genomic DNA as the template. The amplicon was purified using the Gel/PCR DNA Fragments Extraction Kit (Geneaid, Taipei, Taiwan) and directly sequenced using a 3730 DNA analyzer (Applied Biosystems, CA, USA). The sequence analysis was performed using NCBI packages.

**Table 2:** Oligonucleotide primers used in this study.

Name	Sequence (5′→3′)	Size(bps)	Restrictionsite
HP1054-F	CAATCAGGTGTTGTTCCAATTC	1182	
HP1052-R	TCGCATGGACAGCTGAAAGCAA		
minCN	GAATTC GTCATGTTAAAAACGAATC	588	EcoRI
minCC	CTCGAG TTTGCTTCATAATACTTCCTT		XhoI
0405-F	GGATCCCTTACTCAACCCTAA	1400	
0405-R	GGATCCTTAAAAATAGTTTTAGC		
pflaF	TTTATTATAGCCCATTTTCATGCT	127	
pflaR	AGGCCTCCTTGTTATAAAAAACCCA		
FtsZP-F	GAATTC ATGGTTCATCAATCAGAGAT	1158	EcoRI
FtsZP-R	CTCGAG TCAGTCTTGCTGGATTCTCA		XhoI
PminD1-F	GAGCTC AGGAATCATATGGCAATA	807	SacI
PminD2-R	AAGCTT AAAAAAATCAAACAAACTCA		HindIII
minCec-F	GGGATCCATGTCAAACACGCCAAT	696	BamHI
minCec-R	CGTCGACTCAATTTAACGGTTGAA		SalI
NHis-F	ATATACCATGGGCAGCAGCCATCA		

### Construction and Complementation of a *H. pylori minC* Mutant

The *H. pylori minC* was a PCR amplified from the strain NCTC 11637 genomic DNA using primers minCN and minCC. The PCR product was cloned into the SphI site of pUC18 to generate pCPY001. To create an insertional mutant of *minC* in *H. pylori*, the chloramphenicol acetyltransferase (*cat*) cassette form pAV35 (kindly provided by J. M. Ketley) [18] was digested with PvuII and inserted into the unique SphI restriction site 27 bp from the start site of *minC_Hp_* in pCPY001 to generate pCPY002. The chromosomal *minC* locus was disrupted through a homologous recombination upon transforming strain NCTC 11637 with pCPY002. A transformant, selected on BAPs supplemented with Cm, was designated *H. pylori* PY1. The resulting PY1 was confirmed by a PCR analysis using the same primers of minCN and minCC.

To complement *H. pylori* PY1, plasmid pCHL2 was constructed in several steps to be used as a vector. (1) The *hp0405* gene, amplified using PCR with the primers 0405-F and 0405-R, was cloned into the EcoRV site of pOC10 [19] and the fragment between StuI-HincII restriction sites was subsequently removed, which resulted in the vector pOC0405. (2) The *H. pylori* flagella promoter, P*_flaA_*, was amplified using a PCR with primers pflaF and pflaR and ligated with a pTZ57R/t vector to generate pTZ-PflaA. (3) The *kan* cassette of pJMK30 (provided by J. M. Ketley) [18] was cloned into the XbaI site of pTZ-PflaA to generate pTZ-PflaAKm. (4) P*_flaA_* and *kan* were subsequently cloned into the EcoRI site of pOC0405, generating plasmid pCHL2. The obtained pCHL2 vector possessed a unique StuI for cloning genes of interest between P*_flaA_* and *kan*.

Regarding complementation, *minC_Hp_* was PCR-amplified from the *H. pylori* NCTC 11637 genomic DNA, using the primer pair minCN/minCC and was cloned into the unique StuI restriction site of pCHL2 to generate the construct pCPY003. The plasmids were then transformed into mutant PY1 and NCTC 11637 through natural transformation and were selected on BAPs supplemented with Kan. The ectopic integration of the cloned *minC* in the strain PY2 (PY1-complemented strain) was verified with the PCR using the primers minCN and minCC, in which an amplicon of 1.3 kb (the *minC* gene plus a *cat* gene) and 0.6 kb (the *minC* gene only) were obtained. The ectopic integration of cloned *minC_Hp_* in the strain NCTC 11637 was the designated *H. pylori* PY3 and was verified with PCR analysis using a pair of primers pflaF-minCC. *minC_Ec_* was PCR-amplified from pCPY005 using the primer pair NHis-F/minCec-R and cloned into the unique StuI restriction site of pCHL2 to generate the construct pCPY006. Subsequently, the plasmids were introduced into strain PY1 and NCTC 11637 through natural transformation (described above) and transformants were designated *H. pylori* PY2-5 and PY3-1, respectively. Finally, the complemented strains were verified with the PCR analysis using a pair of primers, pflaF-minCec-R.

### Plasmids Construction

The *minC_Hp_* and *ftsZ* gene were amplified by PCR using the genomic DNA of NCTC 11637 as the template, with the primers minCN/minCC and FtsZP-F/FtsZP-R as the primers, respectively. The products were digested with EcoRI and XhoI and cloned into pET30a cleaved with the same enzymes to yield pCPY004 and pCPY007, respectively. The *minD* gene was amplified by the PCR with the primers PminD1-F/PminD2-R and the amplicon was digested with SacI and HindIII. The SacI-HindIII fragment was cloned into pET30a cleaved with the same enzymes to yield pCPY008. Purified MinC_Hp_, FtsZ or MinD proteins from *E. coli* strain BL21(DE3) carrying pCPY004, pCPY007, or pCPY008 were used to raise rabbit anti-MinC_Hp_, anti-FtsZ, or anti-MinD polyclonal antiserum, respectively (Protech Technology Enterprise, Taipei, Taiwan). The *minC_Ec_* gene was amplified by the PCR from *E. coli* MG1655 genomic DNA using the primers minCec-F and minCec-R. The PCR products were digested with BamHI and SalI and ligated with BamHI-SalI-cleaved pET30a to generate pCPY005. The XbaI-XhoI fragments of pCPY004 and pCPY005 were cloned into XbaI-SalI-cleaved pBAD33 to yield pCPY009 and pCPY010, respectively.

### Western Blot Analysis

Bacteria cell liquid cultures were centrifuged at 13,000×*g* for 1 min and cell pellets were washed twice in PBS. Pellets were resuspended in sterile water. The bacterial suspension was sonicated using an ultrasonicator (Model XL, Misonix, Farmingdale, NY) to break the bacteria. Total protein concentrations were determined by using the Bio-Rad Dc protein assay kit on samples diluted 20-fold in water and on BSA standards in the same diluted buffer. The equal amounts of cell protein per lane were mixed with sample buffer (62.5 mM Tris-HCl, pH 6.8 containing 5% 2-mercaptoethanol, 2% SDS, 10% glycerol, and 0.01% bromophenol blue) and heated in a boiling water bath for 10 min. The samples were subjected to polyacrylamide (12%) gel electrophoresis; the protein bands were subsequently transferred onto the polyvinylidene difluoride (PVDF) membranes and probed with rabbit anti-MinC_Hp_ antibodies. A peroxidase-conjugated goat affinity-purified antibody against rabbit immunoglobulin G was used as the secondary antibody (Cell Signaling). Immunoreactive bands were detected using enhanced chemiluminescence (Millipore) and X-ray film. Band intensities on blots were measured using ImageJ version 1.46.

### Motility Assay

Cells of *H. pylori* grown in liquid cultures were stabbed into a soft agar plate containing 0.35% Bacto-agar in Brucella Broth medium and 5% FBS, followed by the incubation of the culture under microaerobic conditions for 3 to 5 days.

### Cell Morphology Analysis

To test the MinC sensitivity of *E. coli*, the plasmid pCPY009 or pCPY010 was introduced into *E. coli* MG1655. Exponentially growing of strain was serially diluted by 10. Then 3 µl cultures from each dilution was spotted on a plate with and without arabinose (Sigma) and incubated overnight at 37°C. To study the effects of overexpression, MinC_Hp_, cells harboring pCPY009 were grown overnight in LB medium at 30°C. An overnight culture was diluted 100-fold in an LB medium supplemented with Cm and grown for 2 h at 30°C. The cell cultures were added at various concentrations (0, 0.002, 0.02, or 0.2%) of arabinose and grown at 30°C for an additional 2 h. The cells were spun down, resuspended in LB medium, mixed 1∶1 with 2% LB agarose, spotted onto a coverslip, and observed under a light microscope.

### Immunoprecipitation

Liquid cultures of *H. pylori* were grown to mid-log phase (OD_600_ = 0.6 - 0.8) and harvested by centrifugation at 6000×*g*. Cell-free extracts were prepared by suspending cells in a sonication buffer (20 mM Tris-HCl, pH 8.0 containing 300 mM NaCl) and sonicated for 1 min with an ultrasonicator. The resultant suspensions were then centrifuged at 13,000×*g* for 20 min at 4°C and the supernatants containing proteins were collected. The supernatants were mixed with 30 µl of 20% (w/v) Protein A Sepharose CL-4B (GE Healthcare) for 1 h to remove nonspecifically bound proteins. An aliquot (50 µl) of supernatant fraction was used as a control for total protein levels prior to immunoprecipitation (IP). Lysate was then incubated with anti-MinC_Hp_, anti-FtsZ, or anti-MinD antibodies coupled to Protein A Sepharose beads and incubated with shaking (40 rpm) at 4°C overnight. Beads containing protein complexes were washed 3 times with IP buffer (0.5% NP-40/PBS) and then boiled in sample buffer for 15 min. Samples were subsequently analyzed by SDS-PAGE, followed by immunoblotting.

### Nucleotide Sequence Accession Number

The nucleotide sequence of the *H. pylori* NCTC 11637 *minC* has been deposited in the GenBank under accession number KC896795.

## Results

### *min* Genes are Present in *H. pylori*, but *minC* is not Clustered with *minD* and *minE*, and Shares a Low Similarity to other Reported Bacteria

**Figure 1:**
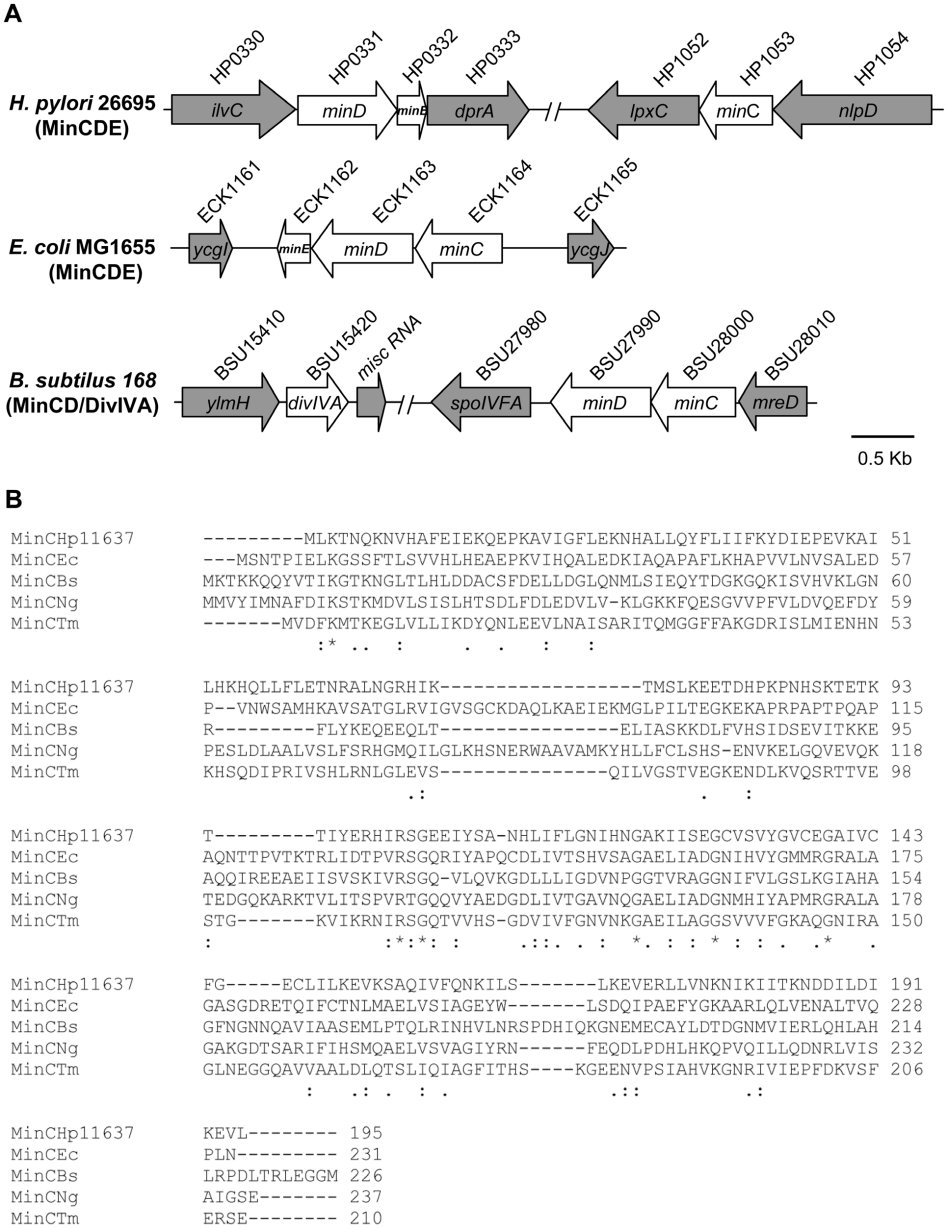
Genomic organization of *min* genes in rod-shaped bacteria. (A) Grey arrows represent the genomic regions surrounding the *min* genes. White arrows show the localization of *min* genes. (B) Sequence comparison of *H. pylori* MinC with those of other bacterial MinC protein. The consensus line below the sequence alignment indicates identity (*), strong conservation (:), and weak conservation (.) of amino acid matches. Organisms in the alignment include *H. pylori* NCTC 11637 (KC896795; Hp11637), *Escherichia coli* (NP_415694.1; Ec), *Bacillus subtilis* (NP_390678; Bs), *Neisseria gonorrhoeae* (YP_208845; Ng), and *Thermotoga maritime* (NP_228853; Tm).

As of March 2013, over 40 strains of *H. pylori* genome have been sequenced and registered in database. Our database search revealed that *minC* is separated from *minD*-*minE* in all sequenced *H. pylori* strains, different from other bacterial genera in which the corresponding genes are clustered [12], [20]. Also, the same genome organization of *min* occurs in all sequenced *Helicobacter* spp. Furthermore, genes flanking *minC* and *minDE* are conserved in all sequenced *Helicobacter* spp.: *nlpD* and *lpxC* flanking *minC* gene and *ilvC* and *dprA* flanking *minDE* (Figure 1A). These findings indicate that these DNA regions are stable.

The *minC*, *minD,* and *minE* genes of *H. pylori* encode proteins of 195 aa (22.38 kDa), 268 aa (29.31 kDa), and 77 aa (8.92 kDa), respectively. MinD and MinE show high degrees of identity in amino acid sequence to those from other bacteria (48.5% and 43.3% identical to *E. coli* and *B. subtilis* MinD, and 29.9% and 14.3% identical to *E. coli* and *B. subtilis* MinE/DivIVA, respectively). However, MinC shares low identity with *E. coli* and *B. subtilis* MinC proteins, 17.4% and 6.2%, respectively. Therefore, this study further investigates the role of MinC in *H. pylori*.

Because of the genetic variability of *H. pylori*, we focused our work on the reference strain NCTC 11637. To investigate the *H. pylori minC*, the gene was cloned from strain NCTC 11637 by a PCR-based strategy using primers HP1054-F and HP1052-R designed according to the *minC* flanking sequences of *H. pylori* strain 26695 (Table 2) for DNA amplification. Sequencing of the amplicon revealed 1,182 bp containing ORF with 195 aa which shared 87% to 95% identity to the MinC from other sequenced *H. pylori* strains and shared 20%, 13%, 13%, and 14% identity with the homologs from *E. coli*, *B. subtilis, Neisseria gonorrhoeae,* and *Thermotoga maritime*, respectively.

It has been shown that four conserved glycine residues at the MinC C-terminus are essential for MinC functionality as a cell division inhibitor and for the interaction of MinC with other Min proteins in *E. coli* [21]. Four glycine residues (G_104_, G_122_, G_129_, and G_139_) were also found at C-terminus of the *H. pylori* MinC proteins. In addition, a lysine residue at the N-terminus (K_3_) and an arginine residue (R_102_) at the C-terminus were also conserved among all known MinC proteins (Figure 1B). These findings suggest that *H. pylori* MinC is a factor involved its cell division, functioning to interact with MinD.

### Mutation of *H. pylori minC* causes Cell Filamentation and Growth and Motility Defects

**Figure 2:**
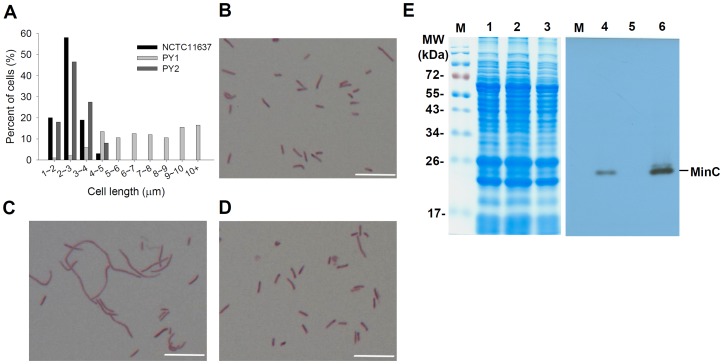
The effect of MinC_Hp_ protein on cell length distribution of *H. pylori*. (A) Cell length distributions of NCTC 11637 (*minC* ^+^), PY1 (*minC* mutant) and PY2 (*minC* complemented strain). (B to D) Gram-stained microscopic images of the three strains shown in panel A to demonstrate the morphology. (B) NCTC 11637; (C) PY1; (D) PY2. Scale bar, 10 µm. (E) SDS-PAGE (left panel) and Western blot (right panel) showing the levels of MinC in strains NCTC 11637, PY1, and PY2. Lanes M, PageRuler prestained protein ladder SM0671 (MBI Fermentas); lanes 1 and 4, NCTC 11637; lanes 2 and 5, PY1; lanes 3 and 6, PY2.

**Figure 3:**
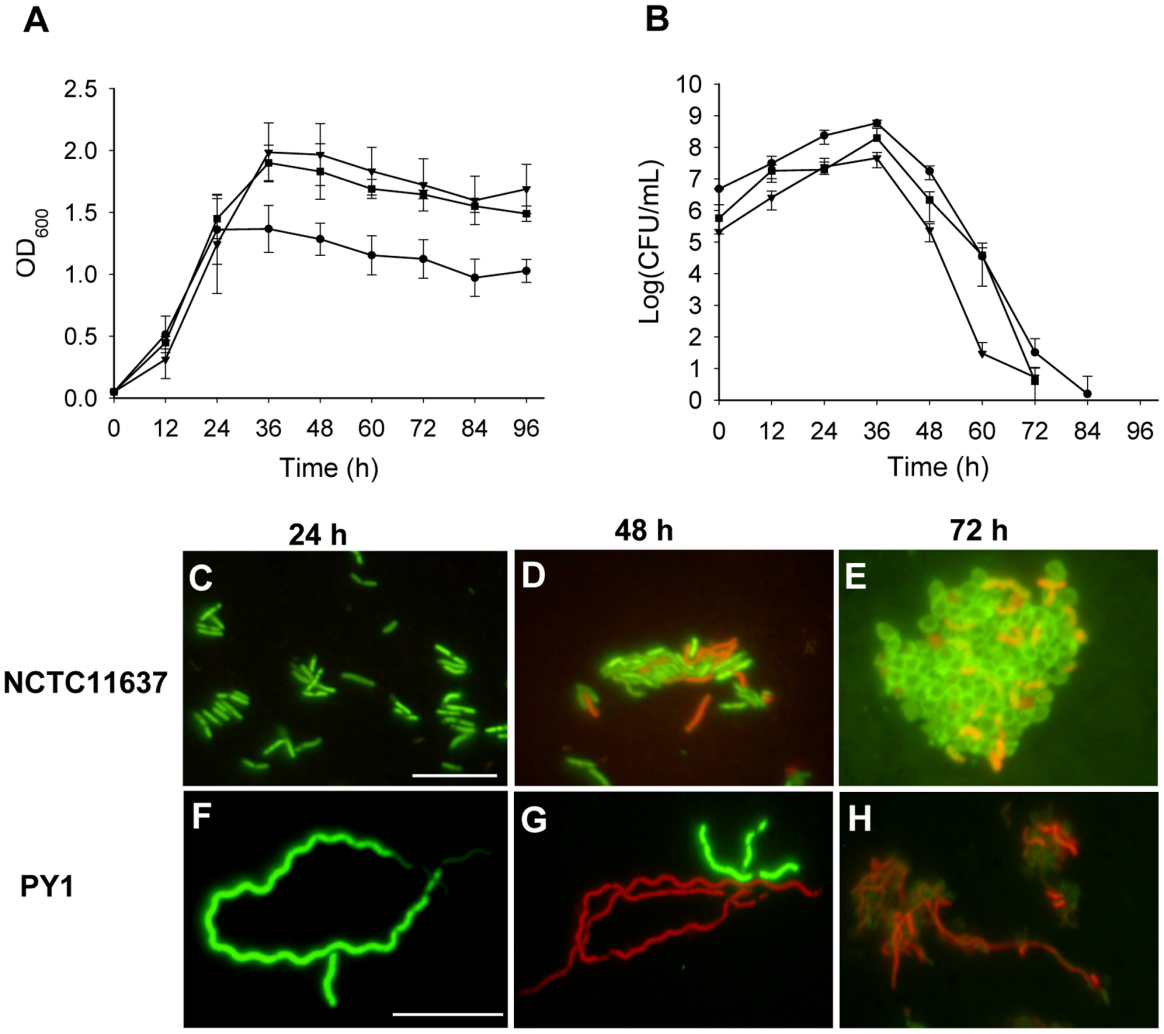
Growth curve, cell viability and fluorescent micrograph of *H. pylori* strains. (A) Cells were grown in Brucella broth medium containing 5% FBS, under microaerobic conditions with gentle shaking at 37°C. Concentrations of the cells were monitored by measuring the turbidity (OD_600_). (B) Viable cells were counted by serially diluted (1∶10) of cultured cells in the fresh medium and plating onto Columbia agar base with 5% defibrinated horse blood. After 3–5 days of incubation, the number of colonies was counted to determine the bacterial proliferation. -•-, NCTC 11637; -▾-, PY1; -▪-, PY2. Error bars represent the standard deviations of the means of triplicate samples. Fluorescent micrograph of NCTC 11637 (C, D, and E) and PY1 (F, G, and H) stained with LIVE/DEAD® kit at 24, 48, and 72 h, respectively, demonstrated morphology and membrane integrity changes during growth in Brucella broth medium containing 5% FBS. Scale bar, 10 µm.

To investigate the role of MinC, a *minC* mutant of NCTC 11637 was constructed by a marker exchange and designated as PY1. Light microscopic observation of PY1 after gram-staining showed cells with different sizes. As shown in Figure 2, the cell length ranged from 1.6 to 25.7 µm with 64.5% of them falling between 5–10 µm. Comparing to that of the wild-type (2.22±0.75 µm), cell elongation of PY1 was obvious. PY1 exhibited a growth rate similar to that of the wild-type, with a generation time of about 6 h (Figure 3A). The OD_600_ of the wild-type reached the maximum (1.34) at 24 h, while that of the mutant increased to a maximum of 1.88 at 36 h. The OD_600_ of both strains declined gradually following cessation of growth. However, the number of viable cells of the wild-type was about 10 times more than that of PY1 at 36 h (5.8×10^8^ versus 4.5×10^7^ CFU/ml), and the amount of cell protein of the wild-type was 1.26 times that of the mutant (3.6 versus 2.85 mg/ml).

**Figure 4:**
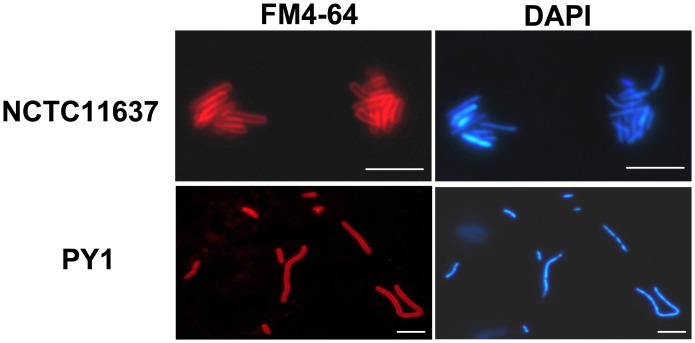
Distributions of DNA and membrane in *H. pylori*. Wild-type and mutant PY1 cells were grown to mid-exponential phase and then stained with DAPI (blue for chromosome) and FM4-64 (red for membrane) and observed by fluorescence microscopy. Scale bar, 5 µm.

To compare the cell morphology and membrane integrity, cells of NCTC 11637 and PY1 grown to 24, 48, and 72 h were stained with LIVE/DEAD® kit and examined by fluorescence microscopy. All the wild-type cells were in rod form till 24 h, a portion of them (ca. 6%) became coccoid form at 48 h, and then almost all cells were in cocoid form at 72 h. In contrast, all PY1 cells maintained the elongated form throughout the experiment (Figure 3). Both strains appeared alive at 24 h; a large portion of the PY1 cells were dead (75%) at 48 h and almost all of them were dead at 72 h. Compared to the mutant cells, larger portions of the wild-type kept alive at 48 h (63%) and 72 h (48%). Furthermore, PY1 contained clearly segregated nucleoids (Figure 4), indicating that mutation in *minC* caused no defects in chromosome replication or segregation.

**Figure 5:**
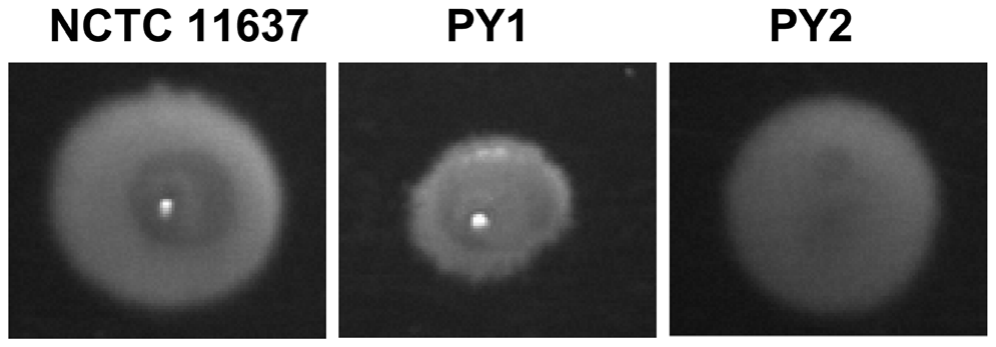
Bacterial motility assay. The indicated strains were stabbed into semisolid agar medium and incubated at 37°C for 72 h.

It is known that cell morphology affects the cell motility in numerous bacteria [22]. As motility of *H. pylori* is crucial in colonizing the gastric mucosa [23], we have evaluated the effects of *minC* mutation on the motility in this study. Tests were performed on a soft agar plate, comparing the area of spreading zones between the *minC* mutant and its parental strain. The results showed that the motility activity is reduced by half in mutant strains. As shown in Figure 5, growth of the wild-type cells resulted in a spreading zone of 15 mm in diameter after 72 h (Figure 5), while that formed by PY1 was about 7 mm in diameter (Figure 5). Since cell division related proteins are not involved in flagellar biosynthesis, it appears that the cellular elongation has reduced the cell motility.

### Reversal of mutant *minC* Phenotype in *H. pylori* by Complementation

To perform the complementation test, we constructed a vector, pCHL2 (Table 1), that allowed for ectopic integration of the plasmid into *H. pylori* chromosome. The integration, targeted at the locus of *hp0405* of *H. pylori*, was shown to cause no detectable effects on the physiology or morphology of *H. pylori* [24], [25]. A complemented strain, PY2, with the *minC_Hp_* gene integrated into the locus of *hp0405* of PY1 was constructed. The expression of MinC_Hp_ in the complemented strain was confirmed by Western blot analysis (Figure 2E). A densitometry analysis indicated that the expression of MinC_Hp_ in PY2 was 1.6 times greater than that of the wild-type NCTC 11637. In addition, about 99% of PY2 cells regained normal cell morphology, exhibited a normal cell length distribution (Figure 2D), and restored their motility (Figure 5). This result suggested that elongation of PY1 cells was significant because of the *minC* mutation and it was not a polar effect on downstream gene expression.

### Cellular Localization of MinC in *H. pylori*

In *E. coli* and *B. subtilis*, MinC is an effector of the Min system responsible for antagonizing cell division and for preventing the sedimentation of FtsZ [11]. However, consequence of *minC* mutation may not be the same for *H. pylori*, because mutation in *minC* gene causes the cell to elongate instead of mini-cell formation observed in *E. coli* and *B. subtilis*.

**Figure 6:**
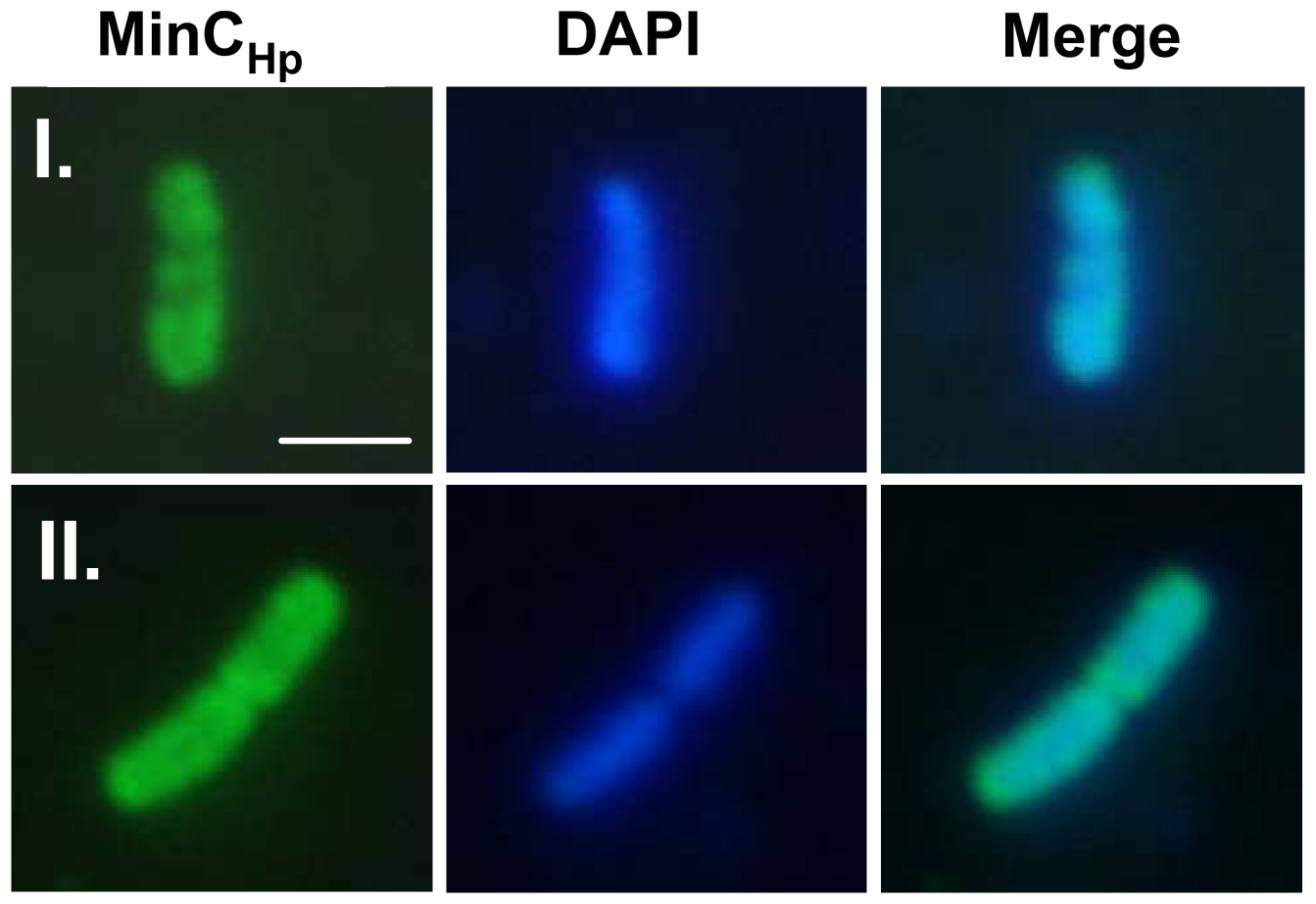
Cellular localization of MinC_Hp_ in *H. pylori*. Selection of cells (I and II) were observed by fluorescence microscopy. IF microscopy was applied using anti-MinC_Hp_ antibody, followed by visualization with FITC-conjugated rabbit IgG. The DNA was labeled by DAPI. Scale bars, 1 µm.

To detect the cellular location of MinC, IF microscopy was performed using antibodies against MinC and a secondary antibody tagged to FITC. The results showed that MinC in the mid-log cells assembled into helix-form structures and located mainly in poles (Figure 6).

### MinC_Hp_ Interacts with MinD but not with FtsZ during Mid-exponential Stage of *H. pylori*

**Figure 7:**
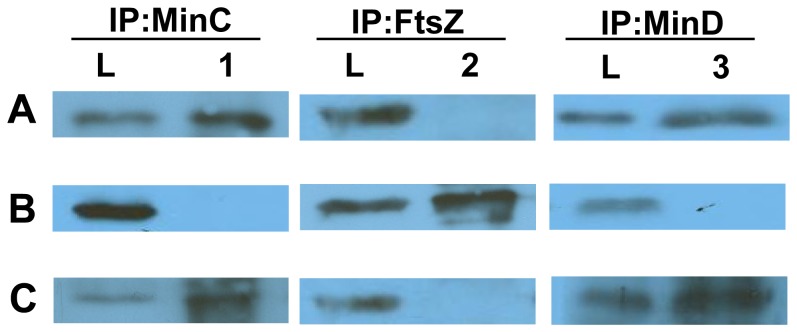
Identification of FtsZ and MinD from co-IP performed with MinC during mid-exponential cultures of NCTC11637. Proteins were eluted after co-IP experiments, samples were separated by SDS-PAGE and detected by Western blotting. Western blots were probed with (A) anti-MinC, (B) anti-FtsZ, and (C) anti-MinD. Lanes L, loading control consisting of whole-cell extract; lanes 1, proteins precipitated with anti-MinC_Hp_; lanes 2, proteins precipitated with anti-FtsZ; lanes 3, proteins precipitated with anti-MinD.

To examine whether MinC interacts with MinD and FtsZ in *H. pylori*, co-IP was performed using antibodies prepared against MinC, FtsZ, or MinD separately, followed by detection of the proteins co-precipitated by Western blotting (Figure 7). Unexpectedly, the MinD protein was precipitated with MinC, but FtsZ was not (Figure 7, lanes 2 and 3).

### The Effects of MinC_Ec_ in *H. pylori*

To test whether MinC of *E. coli* can complement the deficiency in MinC in *H. pylori*, the *E. coli minC* gene was cloned in pCHL2 and introduced into PY1 (forming strain PY2-5) for complementation. Results showed that 81% of the cells had a length shorter than 5 µm with an average of 3.24 µm, demonstrating that MinC_Ec_ could complement the deficiency in MinC_Hp_ in *H. pylori*.

**Figure 8:**
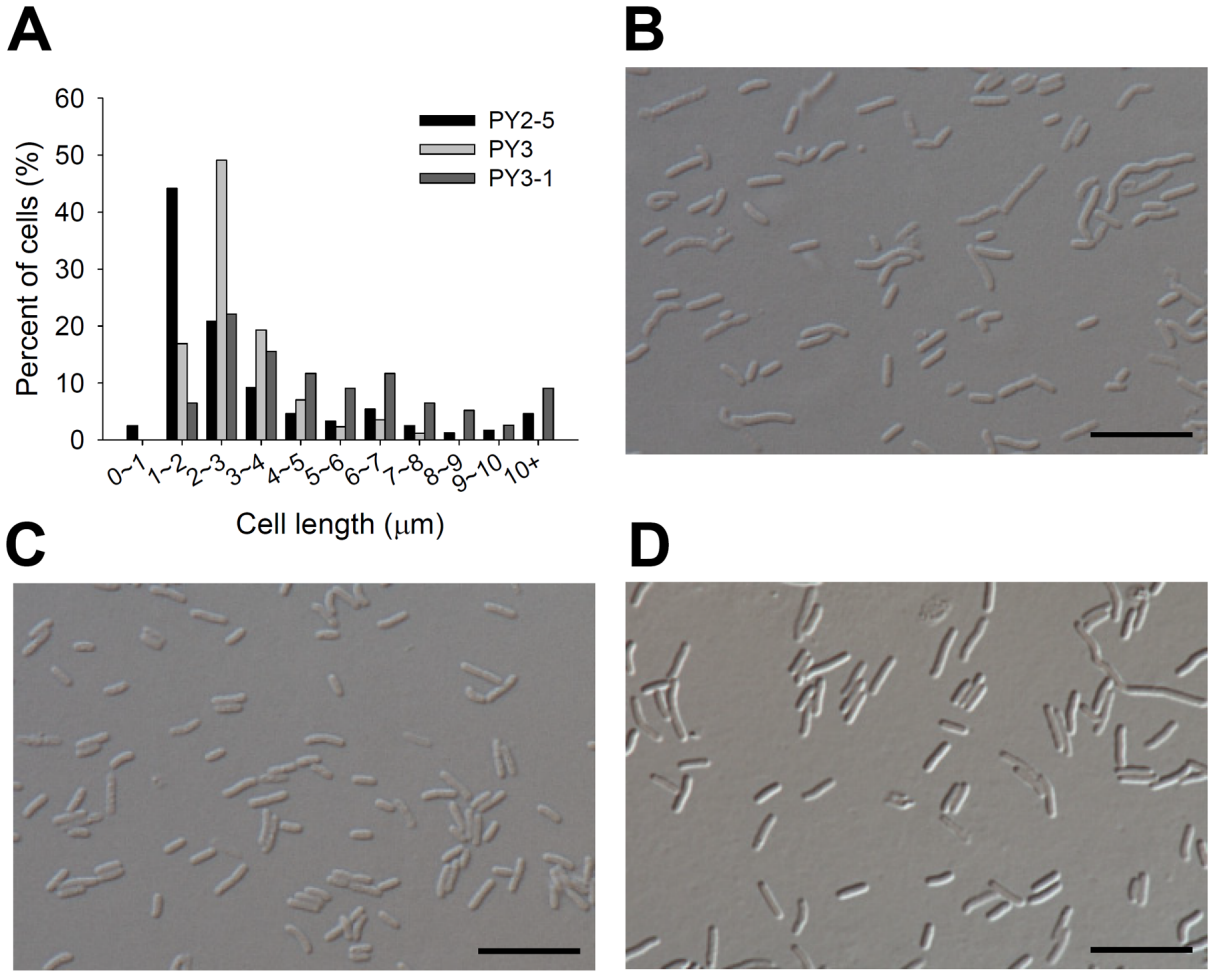
The effects of MinC_Hp_ and MinC_Ec_ proteins on cell length distribution of *H. pylori*. (A) Cell length distributions of the PY2-5, PY3, and PY3-1. (B to D) Differential interference contrast (DIC) microscopic images of the three strains shown in panel A to demonstrate the morphology. (B) PY2-5; (C) PY3; (D) PY3-1. Scale bars, 10 µm.

To inspect the effects of MinC_Hp_ and MinC_Ec_ on *H. pylori* cell division, *minC_Hp_* and *minC_Ec_* were cloned and inserted into the *hp0405* locus of NCTC 11637, resulting in strains PY3 and PY3-1, respectively (Table 1). Cells of PY3 had an average length similar to that of the wild-type (2.99±1.22 µm) and about 7.1% of them were longer than 5 µm (Figure 8A). In contrast, the average cell length of PY3-1 increased to 5.31±3.36 µm (Table 3), and the cells shorter than 2 µm decreased to 7% (Table 3).

**Table 3:** Cell length measurements.

Strain	Genotype	Average cell length ±SD (µm)	Cell shorter than2 µm (%)	Cell between 2 to5 µm (%)	Cell longer than5 µm (%)
*H. pylori*					
NCTC 11637	wild-type	2.58±0.70	17.5	82.5	–
PY1	11637, *minC*::*cat*	7.55±3.86	1.3	26.1	72.6
PY2	PY1, *hp0405*::P*_flaA_-minC_Hp_ kan*	2.77±0.80	15.3	84.2	0.5
PY2-5	PY1, *hp0405*::P*_flaA_-minC_Ec_ kan*	3.24±3.03	46.7	34.6	18.7
PY3	11637, *hp0405*::P*_flaA_-minC_Hp_ kan*	2.99±1.22	16.9	76	7.1
PY3-1	11637, *hp0405*::P*_flaA_-minC_Ec_ kan*	5.31±3.36	6.5	49.3	44.2

### The Effects of MinC_Hp_ in *E. coli*

**Figure 9:**
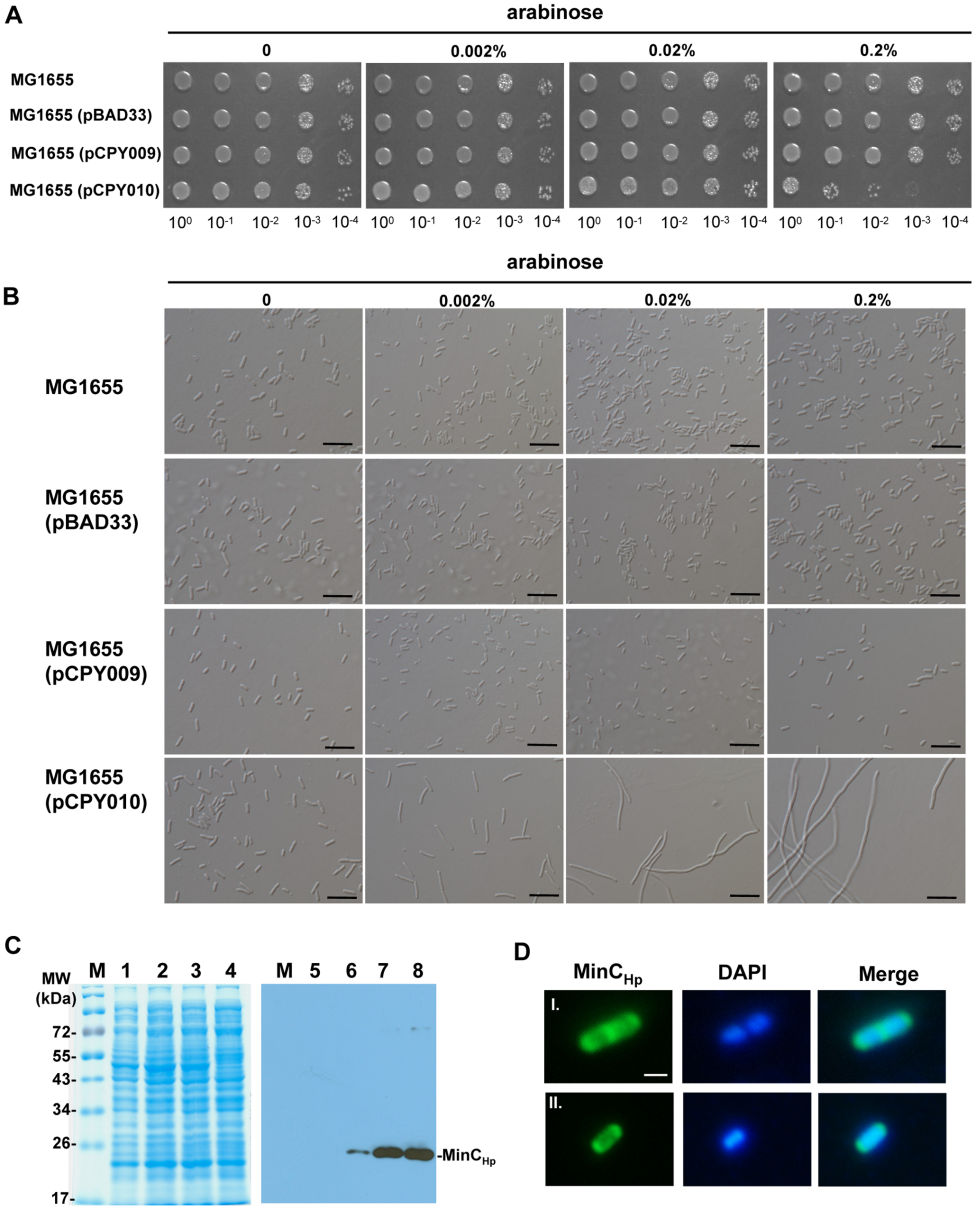
The effects of MinC_Hp_ or MinC_Ec_ on cell viability and morphology of *E. coli*. (A) *E. coli* MG1655 harboring the plasmid pBAD33, pCPY009 and pCPY010, respectively, were serially diluted 10-fold and spotted on LB plates supplemented with the indicated concentration of arabinose at 37°C. (B) DIC micrographs of cells grown in LB with indicated concentration of arabinose at 30°C. Scale bars, 10 µm. (C) SDS-PAGE (left panel) and Western blot (right panel) showing the levels of MinC_Hp_ in MG1655(pCPY009). Cells were grown with 0.002% (lanes 2 and 6), 0.02% (lanes 3 and 7), and 0.2% (lanes 4 and 8) of arabinose, respectively, at 30°C to an OD_600_ of ca. 1.2 for subsequent immunoblot analysis. The MG1655(pCPY009) strain grown without arabinose induction was used as a control (lanes 1 and 5). (D) Selection of cells (I and II) were observed by fluorescence microscopy. IF microscopy to examine the localization of MinC_Hp_ in MG1655(pCPY009). Scale bars, 1 µm.

To inspect the effects of MinC_Hp_ and MinC_Ec_ on *E. coli* cell division, *minC_Hp_* and *minC_Ec_* were cloned in pBAD33 and introduced into the MG1655, resulting in strains MG1655(pCPY009) and MG1655(pCPY010), respectively. As shown in Figure 9A, growth of the MG1655(pCPY010) cells containing *minC_Ec_* gene under control of arabinose-inducible promoter was inhibited by the presence of 0.2% arabinose. But MG1655(pBAD33), carrying the cloning vector only, and MG1655(pCPY009) grew well in the presence of 0.2% arabinose, suggesting that overproduction of MinC_Hp_ was not lethal in *E. coli*.

Light microscopy showed that MG1655 carrying cloning vector only or containing *minC_Hp_* was similar to the wild-type in morphology (Figure 9B), but MG1655(pCPY010) formed filaments in the presence of 0.002% arabinose. In immunoblotting with anti-MinC_Hp_ serum, it was shown that MinC_Hp_ levels were elevated with increased concentrations of arabinose (Figure 9C).

IF microscopy showed that MinC_Hp_ localized at both poles of the *E. coli* cells before septum formation (Figure 9D). Upon septation, the majority of the cells contained intense fluorescence at septum, while some of them still retained small amounts of fluorescence at the poles (Figure 9D), suggesting that MinC_Hp_ also localized at the poles during the late stage of septation in *E. coli.*

## Discussion

In many bacteria, Min proteins are involved in regulation of cell division. It is known that not all three *min* genes are ubiquitously present in all microorganisms and the entire *minCDE* cluster appears to be present only in Gram negative bacteria [26]. In this study, we reveal that *H. pylori* possesses homologs of *minC* and *minDE*, except that they are in two loci. Our sequence analysis here shows that residues conserved in other bacteria are all present in MinC_Hp_ (Figure 1B).

MinC is required for inhibition of septation by FtsZ in many bacteria and deficiency in MinC results in over septation that in turn causes mini-cell formation [8]. In contrast, a MinC deficient mutant of *H. pylori* was found to form elongated cells in this study. Our observations suggest that MinC of *H. pylori* is involved in normal septation that is required for normal cell division. To our knowledge, this is the first report that MinC is required for normal septation instead of inhibiting septation.

In *E. coli*, MinE imparts topological specificity by stimulating MinCD oscillation, thereby ensuring that the concentration of MinCD is highest at the poles [27]. In *B. subtilis*, MinCDJ are localized at the poles or the site of division through polar targeting by DivIVA [28]. In this study, IF microscopy revealed that MinC_Hp_ in the mid-log cells assembled into helix-form structures and located mainly in poles, but do not interact with FtsZ, suggesting that MinC_Hp_-FtsZ interaction is not required for mediation of cell division. It is possible that MinC_Hp_ interacts with other proteins during different stages of cell division in *H. pylori*.

Several studies have shown that Min proteins can function in heterologous background, for examples, the chloroplasts are enlarged when *minC* of *E. coli* is introduced into *Arabidopsis thaliana* [29], cells of *B. subtilis* transformed with *minC* of *E. coli* are elongated [10], *E. coli* cells transformed with *minC* and *minD* of *N. gonorrhoeae* are elongated [13]. In this study, MinC_Ec_ provided in trans resulted in elongation of the wild-type cells and was able to restore the wild-type length to the mutant PY1 (Table 3). It is possible that expression of MinC_Ec_ may prevent the polymerization of FtsZ_Hp_ in *H. pylori*, thereby inhibiting cell division and resulting in cell elongation [26]. In contrast, expression of MinC_Hp_ in *E. coli* did not cause detectable effects on cell morphology. Since the amino acid sequence of FtsZ_Hp_ share high degree of similarity with FtsZ_Ec_ (70%) and MinC_Hp_ exhibited no co-IP reaction with FtsZ_Hp_, it appears reasonable to predict that MinC_Hp_ cannot interact with FtsZ_Ec_. Consequently, MinC_Hp_ could not inhibit the Z-ring formation in *E. coli*. In addition, based on the observations that i) *H. pylori* and *E. coli* FtsZ have different architecture of filaments, ii) the FtsZ-ring positions at both central and acentric regions in *H. pylori,* and iii) daughter cells show considerably different sizes owing to the asymmetrical division of the cells, Specht *et al*. [9] suggest that FtsZ of *H. pylori* possesses a unique intrinsic characteristic different from that of *E. coli* and the cell cycle of *H. pylori* is clearly dissimilar to that of *E. coli*. Thus, the present observations that i) *minC* mutation causes cell elongation instead of mini-cell formation, ii) MinC does not interact with FtsZ, and iii) MinC_Hp_ causes no effects on cell division when expressed in *E. coli* have confirmed and extended the previous findings in *H. pylori* cell division.
